# A modified Kakwani measure for health inequality

**DOI:** 10.1186/2191-1991-2-10

**Published:** 2012-05-06

**Authors:** Mototsugu Fukushige, Noriko Ishikawa, Satoko Maekawa

**Affiliations:** 1 Graduate School of Economics, Osaka University, 1-7, Machikaneyama, Toyonaka, Osaka, 560-0043, Japan; 2 Faculty of Economics, Konan University, 8-9-1, Okamoto, Higashi-Nada-ku, Kobe, Hyogo, 685-8501, Japan; 3 Faculty of Economics, Kansai University, 3-3-35, Yamate, Suita, Osaka, 564-8680, Japan

**Keywords:** Health inequality, Kakwani measure, Decomposition, I14, I10

## Abstract

We propose simple modifications for the Kakwani tax progressivity measure that make it suitable for evaluating access inequality for medical services. Our modification is to measure inequality using the ratio of the concentration index to the Gini coefficient instead of the difference between them. We also propose a measure using the Gini coefficient or concentration index of consumption expenditure as the denominator in the modified measure as an alternative type of modified measure. This measure can also be interpreted as the income/consumption expenditure elasticity evaluated at the mean. Additionally, we propose a decomposition method using expenditure components and provide an empirical example with Japanese data.

## Background

Generally speaking, there are two types of health inequality measures. One is a concentration index of health expenditure ranked by some measure of socioeconomic status. When we adopt household income as a measure of status, this concentration index is said to measure “income-related health inequality.” Another is an application of a measure of progressivity. This measure is usually constructed by taking the difference between the concentration index of health and the Gini coefficient of income.

The former type of measure has simple structures and has been investigated widely by many researchers. LeGrand’s [1] idea, which involved the relationships between socioeconomic groups and health care expenditures, was extended to the concentration index for health expenditure by Wagstaff, van Doorslaer and Paci [2] and Wagstaff, Paci and van Doorslaer [3]. These studies were followed and extended by Wagstaff and van Doorslaer [4] and Koolman and van Doorslaer [5]. Kakwani, Wagstaff and van Doorslaer [6] investigated the statistical properties of the measure and Clarke and Van Ourti [7] investigated the estimation method in the case when income is grouped. Several researchers investigated the factors that affect the measure: Islam et al. [8] studied population aging, and Van Ourti, van Doorslaer and Koolman [9] studied economic growth. Decomposition methods were also proposed by some researchers, e.g., Clarke, Gerdtham and Connelly [10] and Lauridsen et al. [11]. Moreover, to improve or modify the concentration index, several researchers conducted theoretical research. For example, Gerdtham et al. [12] investigated the effects of health rating scales, Wagstaff and Watanabe [13] investigated the relationship between malnutrition, and consumption and wealth, Gravelle [14] removed the effects of age and gender from a concentration index, and Erreygers [15] proposed a new rank-dependent measure.

The latter type of measure is an application of a tax progressivity measure. Tax progressivity measures were first proposed by Musgrave and Thin [16]. Their proposed measure was constructed as follows: divide one minus the concentration index of tax payments by one minus the Gini coefficient of income. Kakwani [17] and Suits [18] also proposed tax progressivity measures. Suits’ modification is similar to our measure. He proposed: one minus the concentration index of tax divied by the Gini coefficient of income as a new measure. However, as for health inequality or inequality of medical expenditures, some researchers have applied the Kakwani measure. For example, Wagstaff, van Doorslaer and Paci [2] proposed this measure as a measure of health inequality and Wagstaff and van Doorslaer [19] provided an interpretation and empirical illustrations. This measure is constructed by subtracting the Gini coefficient of income from the concentration index of medical expenditure, which is also used to measure income-related health inequality. This measure considers the inequality of health expenditure and income distribution simultaneously in evaluating health inequality. This property is advantageous but introduces some difficulties with respect to decomposition into subpopulations. A relatively small number of studies have used this measure. Sutton and Lock [20] applied this measure for analyzing regional differences. Wagstaff and Lindelow [21] and Zhong [22] suggested decomposition methods.

In this paper, we propose a simple modification to the Kakwani measure that maintains its advantages. Our modification creates a custom-made measure of access inequality for medical services. As the original Kakwani measure is a measure of tax progression, it focuses on whether the tax system is progressive, proportional or degressive. When we apply it to health inequality, it cannot determine correctly whether accessibility to the health care system is equal or unequal, where “equal” means the situation where all households or individuals can afford the same level of health care services. We also propose a measure based upon the permanent income hypothesis as an alternative type of the modified measure. The original Kakwani measure is based upon a comparison of the inequalities of expenditures for health care and income. Therefore, it is usually called “income-related” health inequality. However, from an economic perspective, permanent income is preferable to temporal income for measuring household or individual wealth. Deaton and Paxson [23] and Fukushige [24] proposed construction of an inequality measure of consumption. Following their idea, we propose an alternative type of the modified index. This modified measure and its alternative type could also be interpreted as the income or total expenditure elasticity for health care expenditures respectively, using Toyoda’s [25] interpretation. This property is another advantage of our measure.

In the next section, we propose our modifications. In Section 3, we outline the decomposition used in the proposed measure. In Section 4, we summarize Toyoda’s [25] interpretation and present another interpretation of our modified measure. In Section 5, we provide an empirical example to illustrate our modification. Finally, we provide concluding discussions in Section 6.

## Modified measure

Kakwani [17] proposed the difference between the concentration index of tax payments (*C*) and the Gini coefficient of income (G):

(1) K=C−G

as a measure of tax progression. When we consider tax progression, the type of tax system is important. Tax systems can be classified into three types: progressive, proportional and degressive systems. The proportional tax system is a single and central point and this central point is important for this classification. Therefore, whether the difference between the concentration index of tax payments and the Gini coefficient of income is positive, zero or negative is the signal for characterizing the tax system as progressive, proportional or degressive.

When we consider access inequality for medical services, there are two extreme and characteristic situations. One is the case where people pay medical expenditures proportional to their incomes. The other is the case where people pay a constant amount of medical expenditure. When we apply the Kakwani measure for evaluating access inequality for medical services, we cannot characterize these two extreme situations.

In this paper, we propose a simple modification of the Kakwani measure, where the ratio of the concentration index to the Gini index of income is used instead of the difference:

(2) $$\text{MDK}=\frac{C}{G}\text{.}$$

As a ratio, this measure can better characterize the two extreme situations. When people pay medical expenditure proportional to their income, the concentration index of medical expenditure and the Gini coefficient of income are numerically equal and the proposed measure equals one. When people pay a constant amount of medical expenditure, the proposed measure equals zero because the numerator, which is the concentration index of medical expenditure, equals zero. Of course, when the denominator, which is the Gini coefficient of income, is zero, the ratio is undefined, but in empirical studies the Gini coefficient of income is never equal to zero.

Using the proposed measure, we can classify access inequality for medical services into three ranges as follows.

### Degressive

When the concentration index for medical expenditure is negative, the proposed index is also negative. This is a situation where relatively poor people pay more medical expenditure relative to income than do relatively rich people. This is a degressive medical system.

### Accessible

When the concentration index for medical expenditure is positive but less than the Gini coefficient of income, the proposed measure lies between zero and one. In this situation, the ratio of medical expenditure to income decreases with income.

### Less accessible

When the concentration index of medical expenditure is positive and larger than the Gini coefficient of income, the proposed measure is larger than one. In this situation, the ratio of medical expenditure to income increases with income.

By adding two extreme cases, constant payments and proportional payments, we can classify access inequality of medical services into five categories. Table 1 shows these classifications.

**Table 1:** Classification of Access Inequality

**Access inequality**	**Range of MDK**
Degressive	MDK < 0
Constant payment	MDK = 0
Accessible	0 < MDK < 1
Proportional payment	MDK = 1
Less accessible	MDK > 1

When people make medical payments, they, of course, do not obtain utility from making the payment itself. Rather, utility is obtained from health improvement following the medical care. Policy makers have several problems to solve. In an ideal situation, people can access medical services according to their needs. This might be considered a socialist or communist view; however, such a situation cannot exist without imposing costs. In other words, it is impossible.

The less-accessible case can be considered as a case where medical expenditure is a superior good. From a naïve economic point of view, this case applies to the majority of economic goods. However, from a welfare perspective, a situation in which relatively poor people have limited access to medical care should be improved.

In the degressive case, the main concern is that relatively poor people spend a larger proportion of their income on medical care than do relatively rich people. This phenomenon can be assumed to indicate that medical services are easy to access. Why do relatively poor people have more health problems than relatively rich people? Is it caused by their living conditions, lower education or other factors? Policy makers, government or other authorities should investigate the possible reasons.

We also propose an alternative type of the modified measure by introducing the permanent income hypothesis. Deaton and Paxson [23] and Fukushige [24] proposed the construction of an inequality measure based upon consumption expenditure because consumption is a proxy for individuals’ life-cycle utilities. Therefore, we propose the use of the Gini coefficient for consumption expenditure (G_c_) or a concentration index for consumption expenditure (Cc), which leads us to a calculated concentration curve ranked by income instead of the Gini coefficient. We can define the alternative types of the modified Kakwani (MDK) measures as follows:

(3) $$\begin{array}{ccc} MDK_{C}^{*}=\frac{C}{G_{C}} & or & MDK_{C}=\frac{C}{C_{C}} \end{array}\text{.}$$

These measures are based upon life-cycle utility or permanent income, so we can interpret these alternative types as life-cycle inequality measures, while we interpret the formerly proposed measure MDK as an annual inequality measure.

## Decomposition

As the proposed index is in the form of a ratio, decomposing this measure into various expenditure components is equivalent to a decomposition of the numerator. This numerator is the concentration index for medical expenditures, and there are several studies that use Rao’s [26] decomposition method, including Clarke, Gerdtham and Connelly [10]. Using Yao’s [27] notation and expenditure components with suffixes A, B and C, the expenditure share of component in total expenditure for medical services is written as:

(4) $$\begin{array}{cc} w_{f}=\frac{u_{f}}{u}, & f=A,B,C \end{array}$$

where *u*_*f*_ is the average expenditure for component of the whole population and is the average total expenditure for medical expenditures. Then the concentration index is decomposed as follows:

(5) $$C=w_{A}C_{A}+w_{B}C_{B}+w_{C}C_{C}\text{,}$$

and the modified Kakwani measure is decomposed as follows:

(6) $$MDK=w_{A}K_{A}+w_{B}K_{B}+w_{C}K_{C}=w_{A}\frac{C_{A}}{G}+w_{B}\frac{C_{B}}{G}+w_{C}\frac{C_{C}}{G}\text{.}$$

This decomposition is also valid for the life-cycle inequality measure $\begin{array}{ccc} MDK_{C}^{*} & or & MDK_{C} \end{array}\text{.}$

In the case of decomposition by subpopulation, we need to decompose the numerator and denominator simultaneously. Zhong [22] suggested a method for the Kakwani measure. While the Kakwani measure is a form of differences and the decomposition is a weighted average of the differences of the decomposed components of the concentration index and Gini coefficient, the proposed measure is a ratio, so it is difficult to rewrite in an additive form of the decomposed components. This problem remains for future research.

## Another interpretation

According to Toyoda [25], the ratio of the concentration index to the Gini coefficient can be interpreted as an estimate of the income elasticity evaluated at the mean values when the linear regression coefficient is estimated by the instrumental variables method proposed by Durbin [28]. Following Toyoda’s derivation, we derive the estimate as follows. We first set up the linear regression:

(7) $$\begin{array}{cc} E_{i}=\alpha +\beta Y_{i}+\epsilon_{i}, & i=1,2,\ldots ,N \end{array}$$

where *E*_*i*_*andY*_*i*_ are medical expenditures and income, respectively, and i is ordered by income level. We use ordered income as an instrumental variable and estimate the coefficient *β* as follows:

(8) $$\begin{array}{cc} {\hat{\beta }}_{I}\text{=}\frac{\sum_{i}i\left(E_{i}-\bar{E}\right)}{\sum_{i}i\left(Y_{i}-\bar{Y}\right)}, & {\hat{\alpha }}_{I}\text{=}\bar{E}{-\hat{\beta }}_{I}\bar{Y} \end{array}\text{,}$$

where $\bar{E\;}and\bar{Y}$ are the sample mean values of *E*_*i*_*and*Y_*i*_ respectively. This estimation method was proposed by Durbin [28]. Multiplying the ratio of $\bar{E\;}and\bar{Y}$ to ${\hat{\text{β}}}_{\text{I}}$ we can rewrite the equation as follows:

(9) ηE=β^IY¯E¯=∑iiEi-E¯E¯∑iiYi-Y¯Y¯,

while the Gini coefficient for income can be written as:

(10) $$G=\frac{\sum_{i}\left(2i-N-1\right)Y_{i}}{N^{2}\bar{Y}}=2\frac{\sum_{i}i\left(Y_{i}-\bar{Y}\right)}{N^{2}\bar{Y}}\text{,}$$

and the concentration index for medical expenditures can be rewritten as:

(11) $$C=\frac{\sum_{i}\left(2i-N-1\right)E_{i}}{N^{2}\bar{E}}=2\frac{\sum_{i}i\left(E_{i}-\bar{E}\right)}{N^{2}\bar{E}}\text{.}$$

*η*_*E*_ is an estimator of the income elasticity of medical expenditures evaluated at the sample means. When we use total expenditure instead of income, the modified Kakwani measure is interpreted as the total expenditure elasticity for medical expenditures. This Toyoda interpretation is another advantage of the proposed measure. Then, the classification of the medical system proposed in Section 3 is also reinterpreted in terms of the income or total expenditure elasticity. A negative income/total expenditure elasticity means the measure is negative. When the elasticity is larger than one, it means the measure is larger than one.

## Example with Japanese data

**Figure 1:**
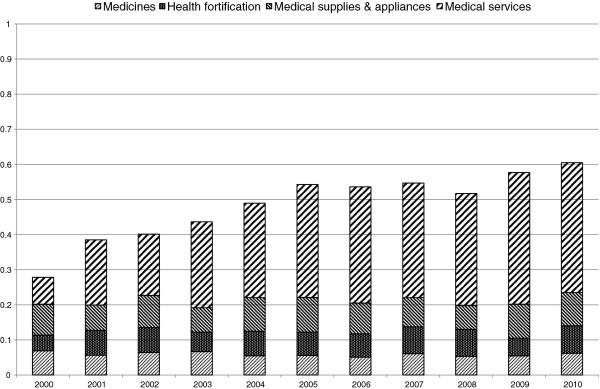
Trend and Decomposition of MDK.

**Figure 2:**
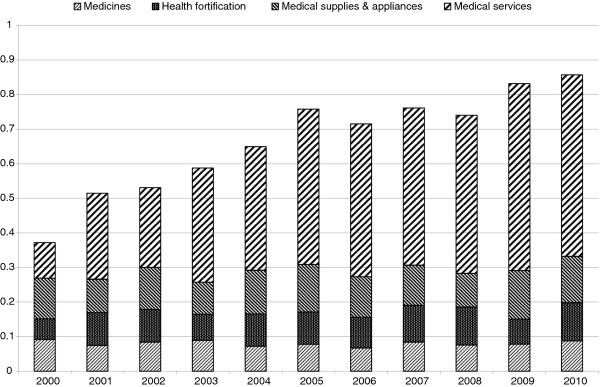
**Trend and Decomposition of MDK**_**C**_

**Figure 3:**
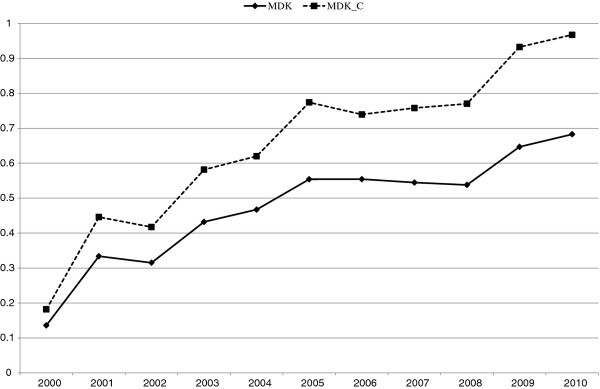
**Trend of MDK and MDK**_**C**_**for Medical Services.**

In this section, we present an empirical example using data from the Family Income and Expenditure Survey in Japan. Because of data availability, we limit our analysis to workers’ households containing two or more persons. Using income quintile data from 2000 to 2010, we calculate the MDK and MDK_C_ of expenditures for medical care and their decomposition into the expenditures for medicine, health fortification, medical supplies & appliances, and medical services. In Figure 1, we show the trend and decomposition of MDK. In Figure 2, we show those of MDK_C_. Both figures illustrate that access inequality for medical services has increased rapidly recently. During this period, the social security system for medical expenditure in Japan has been reformed step by step and patients’ payments have increased. These reforms might affect this increase. Patient payment growth is shown more clearly in Figure 3 where we show the trend in MDK and MDK_C_ for expenditure for medical services. As for MDK_C_ in Figure 3, it starts close to zero in value and approaches one in value. This means that the system of medical service payments was reformed from close to a constant payment system to a proportional payment system. As mentioned above, calculating this inequality measure and comparing it with two extreme cases, we can make the trend in the medical payments system clearer.

**Figure 4:**
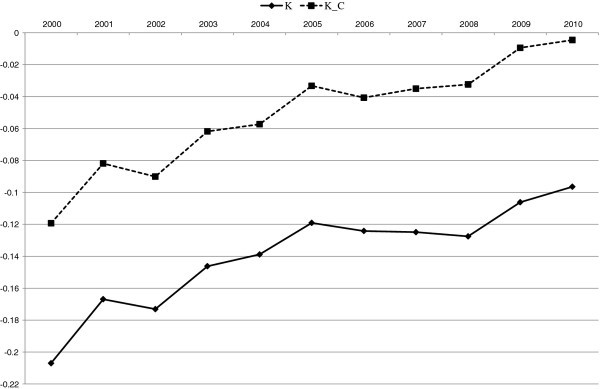
**Trend of K and K**_**C**_**for Medical Services**.

Additionally, to compare the proposed modified measure with the traditional Kakwani measure, we calculate them using the same time periods and present them in Figure 4. K is the traditional Kakwani measure, which is the difference between the concentration index of the medical expenditure and the Gini coefficient of income, and K_C_ is the corresponding measure for expenditure on medical services, which is the difference between the concentration index of medical expenditure and the concentration index of consumption expenditure. Figure 4 shows that the trends, including ups and downs of both the measures (K and K_C_), are very similar to those of the proposed measures (MDK and MDK_C_) and that K_C_ approaches zero, which corresponds to the fact that MDK_C_ approaches one. This means that the system of medical service payments was reformed to a proportional payment system. However, we cannot observe the fact that the system of medical service payments started from close to a constant payment system as suggested by the MDK_C_ in Figure 3. This is an advantage of the modified measure.

## Discussion

In this paper, we proposed simple modifications to the Kakwani tax progressivity measure to make it suitable for evaluating the access inequality of medical expenditures. Our modification was to measure inequality by the ratio of the concentration index to the Gini coefficient instead of the difference between them. We also propose an alternative type: the Gini coefficient or concentration index of consumption expenditure is used as the denominator of the modified measure. We can also interpret the proposed measure as the income/consumption expenditure elasticity evaluated at the mean. This is another advantage of our measure. Additionally, we proposed decomposing the measure into the various expenditure components. Furthermore, an empirical example demonstrated its plausibility and practical usefulness.

Our proposed modification is very simple, useful and practical. However, more empirical evidence is needed to make the advantage of our modification clearer. One of the disadvantages of the proposed measure is the lack of a decomposition method by subpopulation. We will investigate this type of decomposition method in future research. Another disadvantage is that the proposed measure cannot consider reranking by medical expenditures. The reranking problem, which was considered by Bilger [29], is another important problem in evaluating inequality. However, in a developed country such as Japan, the ratio of medical expenditures to income is relatively low and does not introduce a serious reranking problem. Finally, the remaining problem to be solved is to estimate the standard errors of the estimated measure as Kakwani, Wagstaff and van Doorslaer [6] proposed. We will investigate this issue in future research.

## Competing interests

Authors declare that they have no competing interests.

## Authors’ contributions

The authors did the research jointly.
